# First whole-genome analysis of the novel coronavirus (SARS-CoV-2) obtained from COVID-19 patients from five districts in Western Serbia

**DOI:** 10.1017/S095026882100220X

**Published:** 2021-11-02

**Authors:** Dejan Vidanović, Bojana Tešović, Jeremy D. Volkening, Claudio L. Afonso, Joshua Quick, Milanko Šekler, Aleksandra Knežević, Marko Janković, Tanja Jovanović, Tamaš Petrović, Bojana Banović Đeri

**Affiliations:** 1 Veterinary Specialized Institute “Kraljevo”, Kraljevo, Serbia; 2 BASE2BIO, Oshkosh, WI, USA; 3 Institute of Microbiology and Infection, University of Birmingham, Birmingham, UK; 4 Faculty of Medicine, University of Belgrade, Belgrade, Serbia; 5 Scientific Veterinary Institute “Novi Sad”, Novi Sad, Serbia; 6 Institute of Molecular Genetics and Genetic Engineering, University of Belgrade, Belgrade, Serbia

**Keywords:** Coronavirus, COVID-19, SARS-CoV-2, Serbia, whole-genome sequencing

## Abstract

This study was endeavoured to contribute in furthering our understanding of the molecular epidemiology of severe acute respiratory syndrome coronavirus 2 (SARS-CoV-2) by sequencing and analysing the first full-length genome sequences obtained from 48 coronavirus disease-2019 (COVID-19) patients in five districts in Western Serbia in the period April 2020–July 2020. SARS-CoV-2 sequences in Western Serbia distinguished from the Wuhan sequence in 128 SNPs in total. The phylogenetic structure of local SARS-CoV-2 isolates suggested the existence of at least four distinct groups of SARS-CoV-2 strains in Western Serbia. The first group is the most similar to the strain from Italy. These isolates included two 20A sequences and 15−30 20B sequences that displayed a newly occurring set of four conjoined mutations. The second group is the most similar to the strain from France, carrying two mutations and belonged to 20A clade. The third group is the most similar to the strain from Switzerland carrying four co-occurring mutations and belonging to 20B clade. The fourth group is the most similar to another strain from France, displaying one mutation that gave rise to a single local isolate that belonged to 20A clade.

## Introduction

After initially making an appearance in December 2019 [1, 2], the novel betacoronavirus named severe acute respiratory syndrome coronavirus 2 (SARS-CoV-2) swiftly and saliently escalated into an international health emergency, reaching more than 5.6 million cases of coronavirus disease-2019 (COVID-19) by 31 July 2020 [3]. Key elements in revealing the architecture of any emerging pathogen are deciphering its genome and performing a comprehensive phylogenetic analysis. Next-generation sequencing (NGS) techniques were able to rapidly identify SARS-CoV-2, which bore a striking sequence similarity to a bat coronavirus, as well as to the previously described SARS-CoV [1]. SARS-CoV-2 emerged as an enveloped positive-sense single-stranded RNA virus of 29 903 nucleotides (nt) long, encoding for ORF1a [cleaved into 11 non-structural proteins (nsp)] and ORF1b (cleaved into 16 nsp), four structural proteins (spike/S, nucleocapsid/N, membrane/M, and envelope/E) and 11 accessory proteins (3a, 3b, 3c, 3d, 6, 7a, 7b, 8, 9b, 9c and 10) (the International Coronaviridae Study Group 2020, [4, 5]). Accumulation of the SARS-CoV-2 whole genome sequences from the whole world in the GISAID database (https://www.gisaid.org/), and supporting software tools such as Phylogenetic Assignment of Named Global Outbreak LINeages (PANGOLIN) [6], enabled tracking of the geographical prevalence of individual mutations and the paths of virus spread through the human population.

Early GISAID phylogenetic analyses partitioned SARS-CoV-2 based on specific combinations of genetic markers into three variants − A, B and C − distinguishing East Asian isolates from European and American ones [7]. However, this is a dynamic nomenclature, which changes as the virus spreads and mutates, and currently, it distinguishes seven SARS-CoV-2 clades: S, L, V, G, GH, GR, GV and GRY [8]. In parallel, another classification system was developed through the Nextstrain.org platform (https://nextstrain.org/sars-cov-2/), which tracks SARS-CoV-2 evolution in real-time. The Nextstrain classification organises sequences into major clades marked by the year in which the particular SARS-CoV-2 strains have emerged and a letter (i.e. 19A, 19B, 20A, 20B, 20C) [9]. Within each major clade, any new clade is at least two mutations away from its parent major clade, thus smaller clades are being labelled by their parental clade name and specific nucleotide mutations they contain (i.e. 20A/20268G). Nevertheless, both GISAID and Nextstrain classifications are based on dynamic SARS-CoV-2 phylogenetic frameworks, which identify those viral lineages that contribute most to the active spread and those that became inactive.

The first confirmed case of COVID-19 in Serbia was reported on 6 March 2020, with 25 552 more cases registered until 31 July 2020. In Western Serbia, the first official case of COVID-19 was recorded in the district of Cacak on 13 March 2020, in the district of Uzice on 17 March, in the district of Krusevac on 20 March, in the district of Kraljevo on 22 March and in the district of Novi Pazar on 4 April. However, no human isolate-derived sequences from Serbia have been analysed in detail so far. Thus, the objectives of this investigation were to sequence 48 SARS-CoV-2 isolates obtained from COVID-19 patients in five districts in Western Serbia and to determine their phylogenetic relationship to the large database of hitherto published data.

## Material and methods

### Sample collection

During the COVID-19 outbreak in Serbia, the Veterinary Specialized Institute Kraljevo was included in the testing of human naso-pharyngeal swabs for the presence of SARS-COV-2 virus. Samples were taken by the Public Health Epidemiology service from Raska, Rasina, Zlatibor and Moravian districts, all located in Western Serbia (Kraljevo, Novi Pazar, Krusevac, Uzice and Cacak).

### Extraction of total nucleic acids and SARS-CoV-2 qRT-PCR

Total nucleic acids were extracted from samples using a commercial kit, BIOEXTRACT^®^ SUPERBALL^®^, according to the manufacturer's instructions (Biosellal, France). Extractions were performed on a Kingfisher Flex device (Thermo Fisher Scientific, Finland). Extracted RNA was tested immediately and the remaining RNA was stored at −80 °C for further molecular analysis.

Real-time RT-PCR reactions to identify SARS-CoV-2 isolates were carried out using a commercial Real-Time Fluorescent RT-PCR Kit for Detecting SARS-CoV-2 (BGI, China) according to the manufacturer's instructions. Amplification was performed on an AriaMX Real-Time PCR device (Agilent, USA). Ct values were exported and analysed in Microsoft Excel. A total of 48 isolates with Ct between 19 and 25 were selected for further NGS analyses.

### cDNA synthesis and multiplex PCR

cDNA synthesis was performed using SuperScript IV Reverse Transcriptase (Thermo Fisher Scientific, Finland) according to the manufacturer's instructions. Synthesised cDNA was quantified using a Real-Time RT-PCR assay designed by Pasteur Institute, France, targeting the RdRp (RNA-dependent RNA polymerase) gene. The reaction was performed on an AriaMx Real-Time PCR device (Agilent, USA) using Luna^®^ Universal Probe qPCR Master Mix (NEB, USA).

cDNA was amplified using the multiplex ARTIC protocol scheme for SARS-CoV-2 (Version 3), which contains two primer pools with 114 primer pairs. The following reaction was used to set up the multiplex PCR: 2.5 μl template cDNA, 13.15 μl nuclease-free water, 5 μl 5× Q5 reaction buffer (New England Biolabs, USA), 0.5 μl 10 mM dNTPs (New England Biolabs, USA), 0.25 μl Q5 Polymerase (New England Biolabs, USA), 3.6 μl primer pool 1 or 2 (10 μM). Cycling conditions were: 98 °C for 30 s, followed by 30 cycles of 98 °C for 15 s and 65 °C for 5 min, and hold 4 °C indefinitely.

Amplified pools 1 and 2 were combined, cleaned up with 1:1 AmpliClean™ Cleanup kit, magnetic beads (Nimagen, USA), and quantified by Qubit Fluorometer and High sensitivity dsDNA assay (Thermo Fisher Scientific, Finland).

### Nanopore library preparation

Preparation of each library for Oxford Nanopore platform was performed according to the ARTIC protocol using Ligation Sequencing Kit (SQK-LSK109, Oxford Nanopore Technologies) and Native Barcoding Expansion 1–12 (PCR-free) (EXP-NBD104, Oxford Nanopore Technologies). After preparation, 7 ng of the library was loaded on an R9.4.1 flow cell (Oxford Nanopore Technologies), and sequenced on the Oxford Nanopore MinION (Oxford Nanopore Technologies) for 48 h.

### Sanger sequencing

In order to confirm the mutations that were found in sequences obtained on the MinION device, Sanger sequencing was performed covering 37 SNPs using Artic ARTIC Network primers. PCR products were excised from gel and purified using mi-Gel Extraction Kit (Metabion). Sequencing reactions were performed using a BigDye Terminator kit (Applied Biosystems) v3.1 and run on a Genetic Analyzer 3130 (Applied Biosystems).

### Reference-based assembly

Analysis of the MinION raw data was carried out according to the ARTIC nCoV bioinformatics SOP v.1.1.0 (https://artic.network/ncov-2019/ncov2019-bioinformatics-sop.html). Specifically, raw MinION FAST5 traces were basecalled and demultiplexed using Guppy v3.6.0 (GPU build) with the ‘dna_r10.3_450bps_hac’ model and requiring barcodes at both ends, as recommended in the SOP. Determination of consensus genome sequences based on reference read mapping was performed using the ARTIC software tool suite and pipeline (https://github.com/artic-network/fieldbioinformatics) v1.1.3 with the V3 primer set configuration files. Basecalled and demultiplexed reads were filtered using ‘artic guppyplex’ with a minimum read length of 380 nt and a maximum read length of 619 nt. Filtered reads were mapped and consensus-called using ‘artic minion’ in ‘--medaka’ mode (medaka v1.0.3, coverage normalised to 200×).

### Data deposition

Analysed SARS-CoV-2 isolates in five districts in Western Serbia included six isolates from Uzice (all from April 2020), four isolates from Krusevac (all from April 2020), two isolates from Cacak (both from April 2020), 24 isolates from Kraljevo (three from April 2020, two from May 2020 and 19 from July 2020) and 12 isolates from Novi Pazar (one from April 2020, two from May 2020 and nine from July 2020). Genome sequences were deposited in the GISAID database (the Global Initiative on Sharing all Individual Data, available at https://www.gisaid.org) under the accession numbers: EPI_ISL_445086, EPI_ISL_445087, EPI_ISL_454795, EPI_ISL_462435, EPI_ISL_455480, EPI_ISL_462436, EPI_ISL_462437, EPI_ISL_462438, EPI_ISL_482744, EPI_ISL_514425, EPI_ISL_514426, EPI_ISL_514427, EPI_ISL_514428, EPI_ISL_514429, EPI_ISL_514430, EPI_ISL_514431, EPI_ISL_541649, EPI_ISL_541650, EPI_ISL_541652, EPI_ISL_541653, EPI_ISL_541654, EPI_ISL_541656, EPI_ISL_541659, EPI_ISL_541660, EPI_ISL_541661, EPI_ISL_541662, EPI_ISL_582531, EPI_ISL_582532, EPI_ISL_582533, EPI_ISL_582534, EPI_ISL_644568, EPI_ISL_644569, EPI_ISL_644570, EPI_ISL_644571, EPI_ISL_644574, EPI_ISL_644575, EPI_ISL_644576, EPI_ISL_644577, EPI_ISL_644578, EPI_ISL_644580, EPI_ISL_644579, EPI_ISL_644581, EPI_ISL_644582, EPI_ISL_644583, EPI_ISL_582526, EPI_ISL_582527, EPI_ISL_582528, EPI_ISL_582529.

### Phylogenetic analysis

Phylogenetic analysis of 48 SARS-Cov-2 isolates from Western Serbia, the Wuhan reference genome sequence (Wuhan-Hu-1 isolate, NCBI accession number NC_045512.2) and 31 most similar SARS-Cov-2 sequences obtained from other world regions through BLAST search (Supplementary Table S1) was performed in MEGA X software [10] using Neighbour-Joining (NJ) method [11] with a 1000 bootstrap iterations. Additionally, the same analysis was performed using the whole amino acid SARS-CoV2 sequences translated from the whole genome sequences using ExPASy. The Wuhan-Hu-1 complete genome sequence used in this work is identical to the complete genome sequence of the WIV04 isolate (NCBI accession number MN996528.1) that is used by the GISAID database for the detection of the SARS-CoV-2 mutations, with the exception of the 3′ poly-A length. The Nextstrain (https://nextstrain.org/ncov/global) platform was used for the real-time tracking of SARS-Cov-2 genome evolution [12]. In order to reveal the potential impact of nucleotide polymorphisms found in SARS-Cov-2 sequences from Western Serbia at the protein level, the obtained nucleotide sequences were translated into amino acid sequences using the ExPASy translate tool (https://web.expasy.org/translate/).

## Results

We report 48 whole-genome SARS-CoV-2 sequences obtained from Western Serbia patients with coronavirus disease 2019 (COVID-19) sampled in Cacak, Uzice and Krusevac in April 2020 and in Kraljevo and Novi Pazar in the period April 2020–July 2020. The sequences 29 903 nt in length, with a mean read depth ranging from 160 to 220, and harboured 126 distinct single nucleotide polymorphisms (SNPs) in comparison to the first Wuhan complete genome sequence. Four of the detected SNPs occurred in non-translated 5′/3′ regions, 49 were synonymous changes leading to the same amino acid as in the original codon, while 75 of the detected SNPs were deduced to cause amino acid replacements, as summarised in Table 1 and Supplementary Table S2. Thirty-seven of the most common SNPs, including nucleotide positions 241, 1551, 3037, 3169, 4002, 5950, 6267, 9421, 9961, 10265, 10396, 12052, 12525, 13536, 14408, 15720, 15972, 16178, 19648, 19718, 19732, 20178, 23403, 23621, 23674, 23731, 25492, 25966, 26700, 27881, 28077, 28759, 28881–28883, 29477 and 29778, were additionally verified by Sanger sequencing, while the SNP at position 10097 was not examined by Sanger sequencing but was indirectly verified as being present in different SARS-CoV-2 sequences that were sequenced in different laboratories using different sequencing technology.

**Table 1.** Overview of detected SNPs leading to four substitutions in non-translated regions and deduced 75 amino acid changes in 48 whole-genome SARS-Cov-2 isolates from Western Serbia.

Genomic position in the referent genome	Nucleotide in the reference genome Wuhan NC_045512.2	Nucleotide in the samples from Western Serbia	Genome region	Amino acid substitution
219	G	T	5′ UTR	N.A.
241	C	T	5′ UTR	N.A.
1551	C	T	NSP2	249 A > V
1722	C	T	NSP2	306 A > V
2973	C	T	NSP3	85 A > V
3267	C	A	NSP3	183 T > N
4002	C	T	NSP3	428 T > I
4113	C	T	NSP3	465 A > V
5107	A	C	NSP3	796 E > D
5578	G	T	NSP3	953 M > I
5950	G	T	NSP3	1077 K > N
6267	C	T	NSP3	1183 A > V
6566	G	T	NSP3	1283 D > Y
7749	C	T	NSP3	1677 T > I
10028	A	G	NSP4	492 T > A
10097	G	A	NSP5	15 G > S
10105	G	T	NSP5	171 M > I
10265	G	A	NSP5	71 G > S
10762	G	T	NSP5	236 K > N
10874	A	G	NSP5	274 N > D
10965	C	T	NSP5	304 T > I
11083	G	T	NSP6	37 L > F
11113	G	T	NSP6	47 M > I
11851	G	T	NSP7	3 M > I
12026	A	G	NSP7	62 M > V
12052	G	T	NSP7	70 K > N
12525	C	T	NSP8	145 T > I
13424	C	T	NSP10	134 R > C
13793	G	A	NSP12	118 R > H
14371	G	T	NSP12	311 A > S
14408	C	T	NSP12	323 P > L
15705	G	T	NSP12	755 M > I
16178	C	T	NSP12	913 S > L
16733	C	T	NSP13	166 S > L
17678	C	T	NSP13	481 T > M
17864	A	G	NSP13	543 Y > C
18452	C	T	NSP14	138 A > V
18972	G	T	NSP14	311 K > N
19648	G	T	NSP15	10 V > L
19677	G	T	NSP15	19 Q > H
19718	C	T	NSP15	33 T > I
19732	G	T	NSP15	38 V > F
21121	G	A	NSP16	155 G > R
21489	A	C	NSP16	277 K > N
21575	C	T	S	5 L > F
21707	C	T	S	49 H > Y
21717	A	T	S	52 Q > L
21809	G	T	S	83 V > F
22677	C	T	S	372 A > V
23403	A	G	S	614 D > G
23621	G	A	S	687 V > I
24213	C	T	S	884 S > F
24776	G	C	S	1072 E > Q
25249	G	T	S	1229 M > I
25492	A	G	NS3a	34 T > A
25555	G	T	NS3a	55 V > F
25563	G	T	NS3a	57 Q > H
25669	C	G	NS3a	93 H > D
25684	G	T	NS3a	98 A > S
25936	C	T	NS3a	182 H > Y
25966	A	G	NS3a	192 K > E
26111	C	T	NS3a	240 P > L
26529	G	T	M	2 A > S
26700	G	T	M	60 V > L
28057	C	T	NS8	55 A > V
28077	G	T	NS8	62 V > L
28580	G	T	N	103 D > Y
28674	C	T	N	134 A > V
28728	C	T	N	152 A > V
28812	G	T	N	180 S > I
28881	G	T	N	203 R > M
28881&28882&28883	GGG	AAC	N	203 R > K & 204 G > R
28910	A	T	N	213 N > Y
29477	G	T	N	402 D > Y
29511	G	T	N	413 S > I
29717	G	A	3′ UTR	N.A.
29778	G	A	3′ UTR	N.A.

N.A., not-applicable.

Seven isolates that contained unidentified base in the following positions: EPI_ISL_541652: 10105G>N, EPI_ISL_541654: 5184C>N, EPI_ISL_644569: 17350G>N, EPI_ISL_644577: 28093C>N, EPI_ISL_644578: 18028G>N, EPI_ISL_644580: 1141G>N and EPI_ISL_644581: 18788C>N were not stated in the Table in order to avoid false positive polymorphisms prediction.

**Fig. 1.**
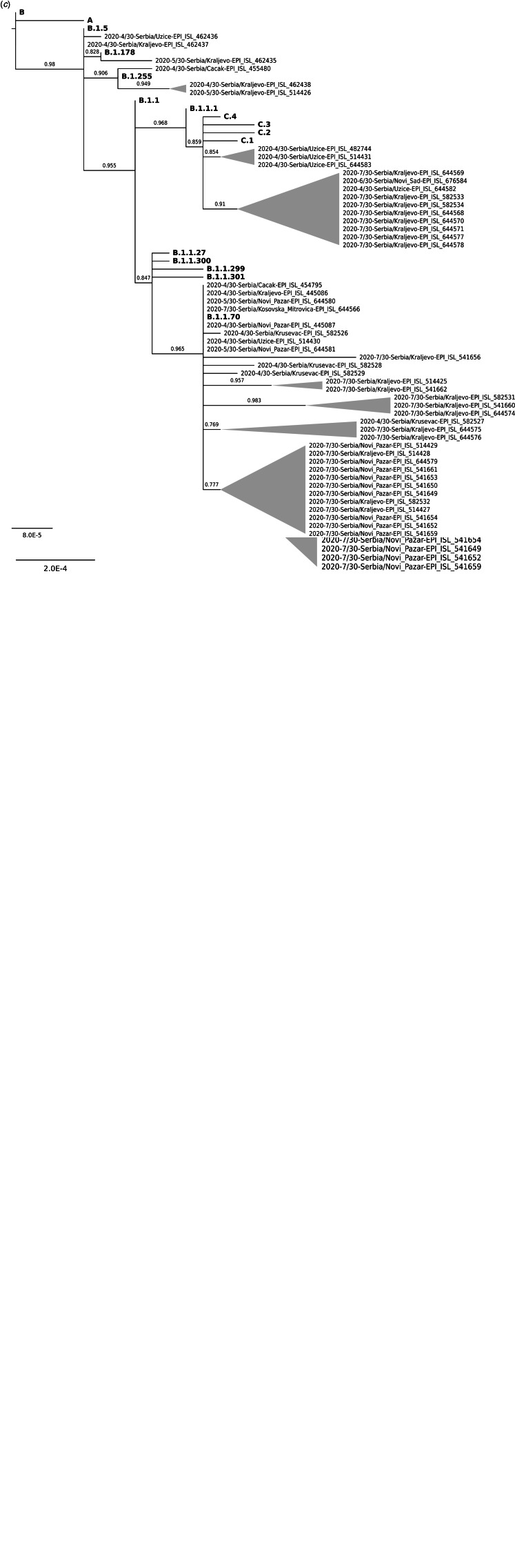
Classification of 48 SARS-Cov-2 isolates from Western Serbia based on GISAID (a) and Nextstrain (b) nomenclature. Phylogenetic analysis of 48 SARS-Cov-2 isolates from Western Serbia (c) in comparison to Wuhan complete genome sequence (NCBI accession number NC_045512.2) and 31 most similar SARS-Cov-2 sequences obtained from other world regions through BLAST search. It involved a total of 79 nucleotide sequences and a total of 29542 positions in the final dataset.

All 48 SARS-CoV-2 sequences from Western Serbia displayed four European major hotspot mutations 241C>T&3037C>T&14408C>T&23403A>G, while 42 out of 48 sequences displayed another major European hotspot mutation 28881GGG>AAC as well. In accordance with presence of the above mentioned four conjoined major mutations along with presence or absence of 28881GGG>AAC, sequences were respectively classified into GR *vs.* G clade, according to GISAID (Fig. 1a), or into 20A *vs.* 20B clade, according to the Nextstrain analysis (Fig. 1b). Six sequences from Western Serbia, collected in April 2020 and May 2020 from Kraljevo, Cacak and Uzice (EPI_ISL_455480, EPI_ISL_462435, EPI_ISL_462436, EPI_ISL_462437, EPI_ISL_462438 and EPI_ISL_514426), characterised by four major hotspots mutations 241C>T&3037C>T&14408C>T&23403A>G as well as by not displaying 28881GGG>AAC mutation, were classified as the clade GR/20A. The remaining 42 sequences from Western Serbia (EPI_ISL_445086, EPI_ISL_445087, EPI_ISL_454795, EPI_ISL_482744, EPI_ISL_514425, EPI_ISL_514427, EPI_ISL_514428, EPI_ISL_514429, EPI_ISL_514430, EPI_ISL_514431, EPI_ISL_541649, EPI_ISL_541650, EPI_ISL_541652, EPI_ISL_541653, EPI_ISL_541654, EPI_ISL_541656, EPI_ISL_541659, EPI_ISL_541660, EPI_ISL_541661, EPI_ISL_541662, EPI_ISL_582526, EPI_ISL_582527, EPI_ISL_582528, EPI_ISL_582529, EPI_ISL_582531, EPI_ISL_582532, EPI_ISL_582533, EPI_ISL_582534, EPI_ISL_644568, EPI_ISL_644569, EPI_ISL_644570, EPI_ISL_644571, EPI_ISL_644574, EPI_ISL_644575, EPI_ISL_644576, EPI_ISL_644577, EPI_ISL_644578, EPI_ISL_644579, EPI_ISL_644580, EPI_ISL_644581, EPI_ISL_644582 and EPI_ISL_644583) collected in April, May and July 2020 from Kraljevo, Cacak, Uzice, Krusevac and Novi Pazar, characterised by five major mutations 241C>T&3037C>T&14408C>T&23403A>G&28881GGG>AAC, were classified as the clade G/20B.

Obtained data suggest that there may have been four independent introductions of the virus. Results of the phylogenetic analysis, presented in Figure 1c, showed that SARS-CoV-2 strains from Western Serbia could be descending from the original Wuhan strain via a more recent intermediate strain from Germany (EPI_ISL_450200 - collected on 28 January 2020), that gave origin to four other intermediate strains that actually entered Western Serbia. The first introduction could have occurred via the strain from Italy (EPI_ISL_542230 – 24 February 2020) that entered Western Serbia in March/April 2020 and contributed to the rise of 31.25–66.67% of local isolates in all five districts by July 2020. This Italian strain carried four major European hotspot mutations 241C>T&3037C>T&14408C>T&23403A>G and shared an identical sequence with Western Serbia isolate from Kraljevo (EPI_ISL_462437 – 23 April 2020). Besides the isolate from Kraljevo, another isolate from Uzice (EPI_ISL_462436 – 28 April 2020) that belonged to 20A clade, has descended from the Italian strain. In parallel, other descendant(s) from this initial strain independently acquired an additional major European mutation 28881GGG>AAC, contributing to 15–30 Western Serbia 20B sequences (the number could not be determined precisely because of the possibility to one to three uncertain secondary introductions from Switzerland and England into this group of sequences, see Discussion). All these sequences displayed four new co-occurring polymorphisms NS5-10265G>A|G71S&NSP12-15972A>G&NSP15-19718C>T|T33I&N-28759T>C, clustering together as one 20B sub-clade and were detected in April 2020 in Kraljevo, Novi Pazar, Krusevac, Uzice and Cacak, and in July 2020 in Kraljevo and Novi Pazar.

The second introduction could have occurred via the strain from France (EPI_ISL_663234 – 9 March 2020) that entered in two districts in Western Serbia – in Cacak (EPI_ISL_455480 – 7 April 2020) and Kraljevo (EPI_ISL_462438 – 22 April 2020 and EPI_ISL_514426 – 11 May 2020), giving rise to 6.25% of local isolates that belonged to clade 20A in April 2020 and May 2020. This strain introduced two novel mutations NSP14-18877C>T&NS3-25563G>T|Q57H into three local sequences, but two of these sequences from Kraljevo also displayed an additional two conjoined mutation NSP14-18471 T>C&N-28910A>T|N213Y that were not carried by introductory strain. Still, these mutations did not show any evidence of spreading in the other three districts in Western Serbia and were no longer detected in July 2020. The third introduction could have occurred via the strain from Switzerland (EPI_ISL_451751- collected on 25 March 2020), carrying four co-occurring polymorphisms NSP3-4002C>T|T428I&NSP5-10097G>A|G15S & NSP12-13536C>T & S-23731C>T, that entered in two districts in Western Serbia, contributed to the rise of 25% of local isolates in these districts by July 2020, but did not show any evidence of spreading in other three districts in Western Serbia. Twelve local isolates descending from this strain created joint 20B sub-clade, that branched further in two subgroups based on displaying an additional new mutation M-26529G>T|D3Y, found in three isolates in Uzice in April 2020 (EPI_ISL_644583, EPI_ISL_482744, EPI_ISL_514431) or displaying two additional new mutations NSP3-5950G>T|K1077N&NSP15-19732G>T|V38F, found in one isolate in Uzice in April 2020 (EPI_ISL_644582) and in eight isolates from Kraljevo in July 2020 (EPI_ISL_644569, EPI_ISL_644571, EPI_ISL_644577, EPI_ISL_644578, EPI_ISL_644570, EPI_ISL_582533, EPI_ISL_582534 and EPI_ISL_644568). Seven of these Kraljevo isolates (all but EPI_ISL_644569) also shared an additional new mutation NSP16-20931G>T. All these additional mutations were locally acquired, independently from the introductory Switzerland strain.

The fourth introduction could have occurred via another strain from France (EPI_ISL_666660 – March 29ht), carrying NSP12-15324C>T mutation that entered in Kraljevo in April/May 2020 and as a single local isolate that was not detected in other four districts in Western Serbia. Even though local 20A strains were no longer recorded in July 2020 in Western Serbia, local 20B strains remained and spread locally, at least in Kraljevo and Novi Pazar, by July 2020.

## Discussion

Nucleotide changes detected in 48 Western Serbia SARS-CoV-2 isolates revealed that genes encoding papain-like proteinase (NSP3), spike protein, N protein, RNA dependent RNA polymerase (NSP12), NS3a protein, 3CL-proteinase (NSP5), endo-RNAse (NSP15), M protein and 3′-to-5′ exonuclease (NSP14) and helicase (NSP13) and trans-membrane NSP4 protein and NSP2 protein, 2′-O-ribose methyltransferase (NSP16), putative trans-membrane NSP6 protein and NSP7, 5′ and 3′ untranslated regions (UTRs) and leader protein (NSP1) and NS8, followed by NSP8 and growth-factor-like protein (NSP10) and NS7b proteins accumulated all detected nucleotide changes, ranging (in descending order) from 14.06% to 0.78% of all detected SNPs, respectively. Regarding the type and positions of the mutations detected in Western Serbia isolates, they are concordant with the reports published so far. The number of transitions was 64.06%, which is agreeable with reports on transitions being generally more frequent in molecular evolution than transversions [13]. Also, finding that C>T nucleotide change is predominant among all of detected mutations (38.28%) is consistent with the other SARS-CoV-2 reports [14–16].

Identified nucleotide substitutions included five major hotspot mutations characteristic for Europe [17–22]. All isolates collected in five districts in Western Serbia contained D614G mutation in spike protein (known to create a more contiguous SARS-CoV-2 form), which was recorded to be introduced in the viral genome for the first time in late February [18] and entered in Western Serbia in March 2020. This is concordant with the first cases of COVID-19 disease reported in Serbia (Subotica, 6^th^ March 2020) and in five districts in Western Serbia: Cacak on 11 March 2020, Uzice on 17 March 2020, Krusevac on 20 March 2020, Kraljevo on 22 March 2020 and Novi Pazar on 1 April 2020. Even though a lack of local SARS-CoV-2 sequences from March 2020 disabled identification of possible earlier introduction(s), obtained the phylogenetic structure of local SARS-CoV-2 isolates and the dates of the local COVID-19 disease case reports suggested at least four independent introductions in Western Serbia.

From four distinct SARS-CoV-2 groups deduced in Western Serbia only descendants of the strains sharing high similarity to the Italian and Switzerland strain remained active by July 2020, while there was no evidence on the activity of the strains sharing high similarity with two French strains beyond May 2020. Four identical SARS-CoV-2 strains found as shared between Italy/Switzerland/France and Western Serbia suggest they were exchanged between countries, but deducing the direction of the introduction requires epidemiological data which are currently lacking. Also, one to three possible additional introductions in Western Serbia could have occurred, but additional data are needed to elucidate if these were introductions (and if so in which direction) or homoplasies. One of these uncertain additional entrances included the isolate from Switzerland (EPI_ISL_500938), carrying NSP3-6566G>T|D1283Y&NSP13-17864A>G|Y543C& S23707C>T&N-29422G>T, which clustered closely with the isolate from Kraljevo (EPI_ISL_541656). Still, since both isolates were sampled on the same date (6 July 2020), and the latter one from Kraljevo harboured four additional mutations NSP3-6566G>T|D1283Y&NSP13-17864A>G|Y543C&S-23707C>T&N-29422G>T, the origin of Kraljevo isolate remained uncertain. Another unclear case was related to two identical isolates from England (EPI_ISL_533103- 15 July 2020 and EPI_ISL_556656 – 18 July 2020), carrying NSP4-9961T>C&NSP12-16178C>T|S913L&NSP15-19648G>T|V10L&NS3a-25492A>G|T34A, which clustered closely with the isolate from Kraljevo (EPI_ISL_582531 – 24 July), but the latter also displayed four additional mutations NSP3-4113C>T|A465V&NSP4-9811&NSP7-12052| K70N&NSP16-20733. Moreover, closely to these isolates, two more samples from Kraljevo (EPI_ISL_541660 – 20 July 2020 and EPI_ISL_644574 – 24 July 2020) clustered, sharing two synonymous NSP3-3169T>C&NSP5-10396G>T mutations and individually acquiring additional different ones. The correct interpretation of the accumulation of numerous different mutations in local isolates in a short time period (based on the collecting dates), require additional data. One more uncertain case involved the strain from Switzerland (EPI_ISL_486532 – 24 June 2020), which, based on the shared mutation M-26700G>T|V60L, clustered closely to eight isolates from Novi Pazar (EPI_ISL_541649, EPI_ISL_541654, EPI_ISL_541661, EPI_ISL_644579, EPI_ISL_541650, EPI_ISL_541653, EPI_ISL_541652 and EPI_ISL_541659) and three isolates from Kraljevo (EPI_ISL_582532, EPI_ISL_514427, EPI_ISL_514428), all collected in July 2020. However, the entire sub-group, in which these isolates clustered together, has descended from a recent unknown intermediate ancestral strain (originating from the Italian strain via local 20A isolate from Kraljevo - EPI_ISL_462437), which also gave the origin to an isolate from Novi Pazar (EPI_ISL_514429 – 14 July 2020) that lacked M-26700G>T|V60L, while displaying additional individual rare mutations.

Taking into account performances of SARS-CoV-2 strains detected in Western Serbia, results suggested that 20A strains did not survive as such in Western Serbia by July 2020, only the local 20B strains did. Regarding mutations that appeared in Western Serbia independently of virus foreign introductions they included NSP5-10265G>A|G71S&NSP12-15972A>G&NSP15-19718C>T|T33I, NSP2-1551C>T|A249V& NSP8-12525C>T|T145I&S-23674A>G&NS8-28077G>T|V62L, S-23621G>A|V687I, NSP4-9421T>C&NS3a-25966A>G|K192E&NS7b-27881C>T and a major triplet mutation N-28881GGG>AAC|R203K&G204R. While some of these polymorphisms were reported to occur in other countries as well, like 10265G>A causing G71S amino acid replacement in 3-CL proteinase, reported as the ninth of ten most frequent mutations detected in the main protease of SARS-CoV-2 [23] and a polymorphic position NS8-28077 which harboured G>C or G>T mutation (which was also reported as positively selected by homoplasy) [24], the others, including amino acid replacement in the spike protein S-23621G>A|V687I that appeared in Kraljevo in July 2020, were not found in public records (except the indirect link in the report by [25] that 23621 position was included in an ‘in frame’ 24 nt deletion in spike protein, informally called ‘Bristol deletion’).

Concerning the impact of all mutations recorded in Western Serbia SARS-CoV-2 strains on the viral performance, their genomic positions support influence on the RNA-RNA, RNA-protein and protein-protein interactions of SARS-CoV-2 affecting strains' performance and possibly virulence. However, epidemiological data on their virulence/pathogenicity/reproduction power are lacking at the moment. This absence of specific epidemiological data on patients with coronavirus disease 2019 linked to sequenced SARS-CoV-2 isolates is one of the shortcomings of the study. Experimental and epidemiological data on various SARS-CoV-2 mutations are just emerging in the literature, in the first place related to mutations affecting the spike protein. Considering information published so far on the impact of amino acid changes in the spike protein matching the ones detected in Western Serbia SARS-CoV-2 strains, beside previously described D614G, they included a signal peptide mutation L5F, which was reported to increase the viral infectivity in four cell culture lines [26], as well as the H49Y mutation, which was reported to increase viral entrance in the cell [27]. Moreover, L5F and D614G were reported to possibly impact CD8T cell epitope production, influencing the severity of infection [28]. On the other hand, the M1229I mutation, occurring in the hydrophobic transmembrane domain of the spike protein, was only considered as co-occurring to several other mutations in the lineage B.1.1.298 (cluster 5), which was characterised as a variant of concern [29]. Thus, while collecting future novel data, at least the Western Serbia strains containing the aforementioned mutations should be followed through time.

The great efforts worldwide are being put forward to sequence as many complete genomes of SARS-CoV-2 as possible, allowing a comprehensive viral phylogeographic study, and more recently they were accompanied by efforts to elucidate viral transcriptome and epitranscriptome as well [5]. However, the same efforts are needed to be put forward to build a comprehensive epidemiological database on patients with coronavirus disease 2019 that will be linked to sequenced SARS-CoV-2 isolates. Only in this way, the entirety of collected data will enable association studies of viral genotype with the viral phenotype (pathogenicity, viability, etc.) and epidemiological data, enabling the development of different knowledge-based strategies to handle this new virus. Given that any of the evolutionary prediction models we are building is as good as the starting datasets and underlying bioinformatics algorithms are, securing a critical amount of relevant data is essential for any credible prediction. Besides mutations leading to amino acid replacements, synonymous nucleotide changes may also contribute to different viral pathogenicity levels, as was already shown for another positive-sense single-stranded RNA virus [30]. Therefore, all detected nucleotide changes, even the silent ones, should be taken into account when conducting data mining analysis and viral genotype−phenotype association SARS-CoV-2 studies. Such knowledge would be extremely valuable in developing efficient SARS-CoV-2 monitoring/novel fast screening tests, devising novel strategies to fight the virus and choosing adequate medical nurture for patients with coronavirus disease 2019.

## Data Availability

The sequences of SARS-CoV-2 that are subject of this study are deposited in the Global Initiative on Sharing all Individual Data (GISAID), available at https://www.gisaid.org.in EpiCoV database. It can be accessed under numbers described in the Material and methods section, data deposition. Supplementary Table S1 is available at Epidemiology & Infection online.

## Supplementary material

For supplementary material accompanying this paper visit http://dx.doi.org/10.1017/S095026882100220X. click here to view supplementary material
